# Protocol for precise signal synchronization of electrophysiology, videography, and audio recordings using a custom-made pulse generator

**DOI:** 10.1016/j.xpro.2023.102306

**Published:** 2023-05-12

**Authors:** Sarah Dagher, Shimpei Ishiyama

**Affiliations:** 1 Universitätsmedizin der Johannes Gutenberg-Universität Mainz, Institut für Pathophysiologie, 55128 Mainz, Germany

**Keywords:** Biophysics, Model Organisms, Neuroscience, Behavior, Systems Biology, Biotechnology and Bioengineering

## Abstract

Precise signal synchronization is vital for accurate analysis in systems neuroscience. Here, we present a protocol for synchronizing electrophysiology, videography, and audio recordings using a custom-made pulse generator. We describe steps for building the pulse generator, installing software, connecting devices, and running experimental sessions. We then detail signal analysis, temporal alignment, and duration normalization. This protocol offers flexibility and cost-effectiveness, addressing limited shared knowledge and providing a solution for signal synchronization in various experimental setups.

## Highlights

• Protocol for synchronizing electrophysiology, audio, and two-camera video recordings
• Steps to build a custom low-cost pulse generator for signal synchronization
• Details on setting up the pulse generator, running experiments, and signal analysis
• Adaptable synchronization concept for diverse research settings in systems neuroscience

## Abstract (editor highlights)

Publisher’s note: Undertaking any experimental protocol requires adherence to local institutional guidelines for laboratory safety and ethics.

## Graphical abstract

**Figure:**
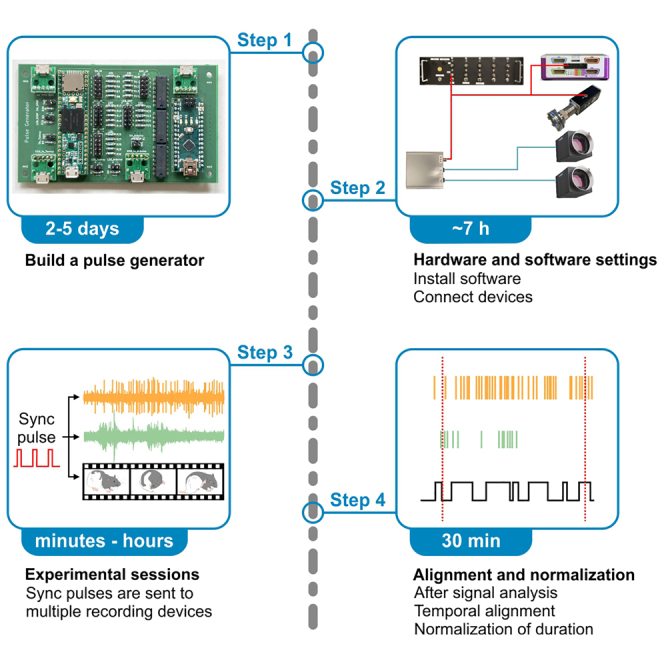

## Before you begin

Experiments in systems neuroscience often involve recording a variety of signals including electrophysiology, neural imaging, behavioral videography, audio, operant behaviors, and stimulus paradigms. Synchronization of these signals captured by multiple devices is crucial for accurate data analysis and application for closed-loop experiments. While commercially available recording systems offer multimodal recording with integrated synchronization, custom setups using individually purchased devices can offer greater flexibility at a lower cost. For example, users can choose a camera with specific features desired for a particular experiment. However, custom setups require users to establish a signal synchronization environment, since comprehensive guidance on connecting and synchronizing various devices is often lacking. By sharing our synchronization protocol, we aim to help a broad range of experimental neuroscientists build or improve their experimental infrastructure without the significant hurdles that many labs, including our own, have faced. This knowledge will empower researchers to make informed decisions when setting up their labs and facilitate more efficient and cost-effective solutions for signal synchronization in systems neuroscience.

Typically, signal synchronization is achieved by sending transistor-transistor logic (TTL) digital pulses to each device, followed by temporal alignment of signals later in the analysis, much like how clapper boards are used to synchronize sound and vision in filming. In this article, we introduce our custom-designed low-cost pulse generator and explain how to synchronize signals in electrophysiology, audio, and video recording using a setup with two cameras. Our specific hardware and software are used as examples, but the synchronization concept can be applied to most research settings.

### Software installation

**Timing: 1 h**

In this section, we install necessary software (see key resources table). Software settings will be explained in “step-by-step method details” sections.

1. Install MATLAB.
2. Install Arduino-related software.
  a. Install Teensyduino.
  b. Download pulseGenerator folder from https://github.com/neurogelotology/synchronization_protocol/tree/main/Pulse_Generator_PCB/
  c. Download Pulse_Generator_software folder from https://github.com/neurogelotology/synchronization_protocol/tree/main/Pulse_Generator_software.
  d. Install Arduino IDE.
  e. Install Support for Arduino Hardware in MATLAB.
3. Download and install Spinnaker SDK with a driver from FLIR.
  a. Use “Application Development” option.
  b. Enable “DirectShow” under “Third Party Support”.
  c. Search “SpinnakerDirectShow…dll” under “C:∖Program Files∖Point Grey Research∖Spinnaker” in case of Windows OS.
  d. Run Command Prompt as administrator and go to the directory where the .dll file is located.
  e. Register the .dll file by typing “regsvr32 SpinnakerDirectShow_v140.dll” (the .dll file name may differ)
4. Install the USB3.1 interface card sold by FLIR.
5. Download and install the bonsai software.
6. Close SpinView if opened.
7. Open bonsai and click on “Manage Packages”.
8. Click on “Installed packages” at the top left to see currently installed libraries.
9. The following libraries need to be installed. To install a library, click on “Online” at the top left, and type the library name in the top right box.
  a. Spinnaker Library.
  b. Bonsai Vision Library.
  c. Bonsai Dsp Library.
  d. Bonsai Core Library.
10. Install Avisoft-RECORDER USGH (for operating the UltraSoundGate microphones) or Avisoft-RECORDER (for third-party acquisition devices) from Avisoft.

### Prepare pulse generator materials

**Timing: Depends on purchase speed**

We prepare materials including the printed circuit board (PCB) to build a custom pulse generator.

11. Order necessary materials (see “materials and equipment”).
12. Outsource the PCB for printing (PCB file available at https://github.com/neurogelotology/synchronization_protocol/tree/main/Pulse_Generator_PCB/pulseGenerator

***Note:*** We provide the PCB in KiCad files. KiCad is an open-source electronics design software, allowing users to freely edit the circuit if necessary. PCB printing services, such as aisler.net, accept KiCad files.

### Institutional permissions

The example audio data provided in this protocol were generated using a male Long-Evans rat. All experimental procedures were performed according to German guidelines on animal welfare under the supervision of local ethics committees in accordance with the animal experimentation permit (G 20-1-082).

## Key resources table

**Table:** 

REAGENT or RESOURCE	SOURCE	IDENTIFIER
**Deposited data**		

PCB design files for the pulse generator	This paper	https://github.com/neurogelotology/synchronization_protocol/tree/main/Pulse_Generator_PCB/pulseGenerator
Example data files containing sync TTL times	This paper	Mendeley Data: https://doi.org/10.17632/98rzkwy8s6.1

**Experimental models: Organisms/strains**		

Long-Evans rat (RjOrl:LE), 5-week old, male	Janvier Labs	https://janvier-labs.com/en/fiche_produit/long-evans_rat/

**Software and algorithms**		

Arduino IDE	Arduino	https://www.arduino.cc/en/software
Teensyduino	PJRC	https://www.pjrc.com/teensy/td_download.html
MATLAB	MathWorks	https://www.mathworks.com/products/matlab.html
Support for Arduino Hardware	MathWorks	https://www.mathworks.com/help/supportpkg/arduinoio/ug/intro.html
Spinnaker SDK (incl. SpinView)	FLIR	https://www.flir.com/products/spinnaker-sdk/
bonsai	Bonsai Foundation CIC	https://bonsai-rx.org/
Cheetah (electrophysiological recording)	Neuralynx	https://neuralynx.com/software/cheetah
Avisoft RECORDER USGH	Avisoft	https://www.avisoft.com/downloads/
Pulse generator Arduino Sketch	This paper	https://github.com/neurogelotology/synchronization_protocol/tree/main/Pulse_Generator_software
MATLAB functions for the pulse generator	This paper	https://github.com/neurogelotology/synchronization_protocol/tree/main/Pulse_Generator_software
MATLAB functions to extract sync TTL times from each signal	This paper	https://github.com/neurogelotology/synchronization_protocol/tree/main/syncTTL
bonsai file	This paper	https://github.com/neurogelotology/synchronization_protocol/tree/main/Bonsai

**Other**		

LabLynx (electrophysiology data acquisition system)	Neuralynx	https://neuralynx.com/hardware/lablynx-portable-system
UltraSoundGate 116Un (ultrasound microphone)	Avisoft	https://www.avisoft.com/ultrasoundgate/116un/
BFS-U3-28S5C-C (camera)	FLIR	https://www.flir.de/products/blackfly-s-usb3/?model=BFS-U3-28S5C-C&vertical=machine+vision&segment=iis
PCB print service	github.com/neurogelotology	PCB files: https://github.com/neurogelotology/synchronization_protocol/tree/main/Pulse_Generator_PCB/pulseGenerator Printing: Seeedstudio; Aisler
Rack mount housing 3U	Thomann	Adam Hall 87409V Rack Housing 3U
M3 standoff spacer	Mouser Electronics	https://eu.mouser.com/ProductDetail/Wurth-Elektronik/971100321?qs=wr8lucFkNMX9V0JHOUB5HA%3D%3D
M3 nuts	Mouser Electronics	https://eu.mouser.com/ProductDetail/Essentra/04M030050HNDIN34814?qs=T3oQrply3y%252BoX1ymaXFOZA%3D%3D
M3 screws	Mouser Electronics	https://eu.mouser.com/ProductDetail/PEM/FH-M3-6ZI?qs=l4Gc20tDgJL1BX%2FEwN55AQ%3D%3D
Teensy 3.5 with pin headers	PJRC	PJRC
Arduino Nano 3 with pin headers	Arduino	https://store.arduino.cc/products/arduino-nano
Panel mount white LED	RS	https://uk.rs-online.com/web/p/panel-mount-indicators/7239485
Panel mount green LED	RS	https://uk.rs-online.com/web/p/panel-mount-indicators/0206867
Panel mount momentary push button	RS	https://uk.rs-online.com/web/p/push-button-switches/6903255
Panel mount momentary push button with red LED	RS	https://uk.rs-online.com/web/p/push-button-switches/1116531
Panel mount rocker switch with green LED	RS	https://uk.rs-online.com/web/p/rocker-switches/2400761
Panel mount BNC female connector with GND connector	RS	https://uk.rs-online.com/web/p/coaxial-connectors/5464904
Panel mount USB socket (USB-B outside; USB-A inside)	RS	https://uk.rs-online.com/web/p/usb-connectors/1246393
SN74HC32N OR-gate	RS	https://uk.rs-online.com/web/p/logic-gates/1000761
Resistor 10 kOhm, 5 mm footprint width	Reichelt	https://www.reichelt.com/pl/en/thin-film-resistor-axial-0-4-w-10-kohm-1--vi-mba02040c1002-p233622.html?&trstct=pol_0&nbc=1
Resistor 220 Ohm, 5 mm footprint width	Reichelt	https://www.reichelt.com/pl/en/thin-film-resistor-axial-0-4-w-220-ohm-1--vi-mba02040c2200-p233634.html?&trstct=pol_0&nbc=1
USB micro to USB mini B cable	Reichelt	https://www.reichelt.de/de/en/8in-micro-usb-to-mini-usb-otg-cable-m-m-st-umusbotg8in-p280342.html?&trstct=pos_5&nbc=1
USB micro to USB micro cable	Reichelt	https://www.reichelt.de/de/en/8in-micro-usb-to-micro-usb-otg-cable-m-m-st-uuusbotg8in-p280362.html?&trstct=pos_2&nbc=1
USB-A to USB micro cable	Reichelt	https://www.reichelt.de/de/en/usb-2-0-cable-usb-a-connector-to-micro-usb-b-connector-15-cm-value-11998751-p321478.html?&trstct=pos_10&nbc=1
USB-A / USB-B cable	Reichelt	https://www.reichelt.de/de/en/usb-2-0-cable-usb-a-to-usb-b-connector-1-8-m-roline-11028802-p288316.html?&trstct=pos_2&nbc=1
USB micro B breakout board	Reichelt	https://www.reichelt.de/de/en/development-boards-breakout-board-with-micro-usb-2-0-debo-microusb-2-p240686.html?PROVID=2788&&r=1
Pin headers	Reichelt	https://www.reichelt.de/de/en/pin-header-2x34-pin-bkl-10120312-p280520.html?&trstct=pos_13&nbc=1
Jumper wire cables female to female	Reichelt	https://www.reichelt.de/de/en/developer-boards-jumper-cable-40-poles-f-f-40-cm-debo-kabelset20-p340345.html?&trstct=pol_6&nbc=1
BNC cable	Reichelt	https://www.reichelt.de/de/en/bnc-video-cable-bnc-male-bnc-male-1-0-m-black-n-cvgp01000bk10-p241636.html?&trstct=pos_1&nbc=1
2.5 mm TS cable	Reichelt	https://www.reichelt.de/de/en/nf-cable-jack-2-5-mm-mono-straight-1-8-m-nfke-mg-25-p109572.html?PROVID=2788&gclid=CjwKCAjw3POhBhBQEiwAqTCuBlkCckJCcUvmzDOhtYbK7gBGZnaFfJ47gwFbVXmZkoDCRQjn3QmzvRoC5jYQAvD_BwE&&r=1
GPIO cable (Hirose HR10)	FLIR	https://www.flir.com/products/hirose-hr10-6-pin-circular-connector/
Mini din6 female connector	Jameco	https://www.jameco.com/z/A170718LH-Jameco-Valuepro-6-Terminal-Circular-Mini-DIN-Female-Panel-Mount_2246531.html
Mini din6 male (PS/2) cable	Reichelt	https://www.reichelt.de/de/en/ps-2-cable-plug-to-plug-3-m-roline-11015830-p321073.html?PROVID=2788&gclid=CjwKCAjw3POhBhBQEiwAqTCuBlDGnvMgVUa4XVVTVPXAJK7ocdRZ6JwUYasSvA-EOUV0GLZ5mUM26hoC424QAvD_BwE&&r=1
Multimeter	Conrad	https://www.conrad.de/de/p/voltcraft-vc130-1-hand-multimeter-digital-cat-iii-250-v-anzeige-counts-2000-1090519.html

## Materials and equipment

**Table:** Materials required to build a pulse generator

Material	Quantity	Purpose
PCB print service	1	Circuit board
Rack mount housing 3U	1	Housing
M3 standoff spacer	4	Holding PCB in housing
M3 nuts	4	Fixing spacers in housing
M3 screws	4	Fixing PCB on spacers
Teensy 3.5 with pin headers	1	Main processor
Arduino Nano 3 with pin headers	1	MATLAB triggers
Panel mount white LED	5	Output indicators
Panel mount green LED	5	Input indicators
Panel mount momentary push button	5	Push button triggers
Panel mount momentary push button with red LED	1	Stop button
Panel mount rocker switch with green LED	2	Power switches
Panel mount BNC female connector	10	Outputs and external inputs
Panel mount USB socket (USB-B outside; USB-A inside)	2	Interfacing pulse generator and PC
SN74HC32N OR-gate	3	OR logic gate
Resistor 10 kOhm, 5 mm footprint width	18	Pull-down resistors for switches
Resistor 220 Ohm, 5 mm footprint width	13	For LEDs
USB micro to USB mini B cable	1	Connect USB breakout to Arduino Nano inside the housing
USB micro to USB micro cable	1	Connect USB breakout to Teensy inside the housing
USB-A to USB micro cable	2	Connect panel mount USB socket to USB breakout inside the housing
USB-A / USB-B cable	2	Connect pulse generator to PC
USB micro B breakout board	4	Power switches
Pin headers	90 pins	Connectors on the PCB
Jumper wire cables female to female	62	Connecting pin headers with LED, switches, buttons, or BNC connectors

## Step-by-step method details

### Building a pulse generator

**Timing: 2–5 days**

**Figure 1:**
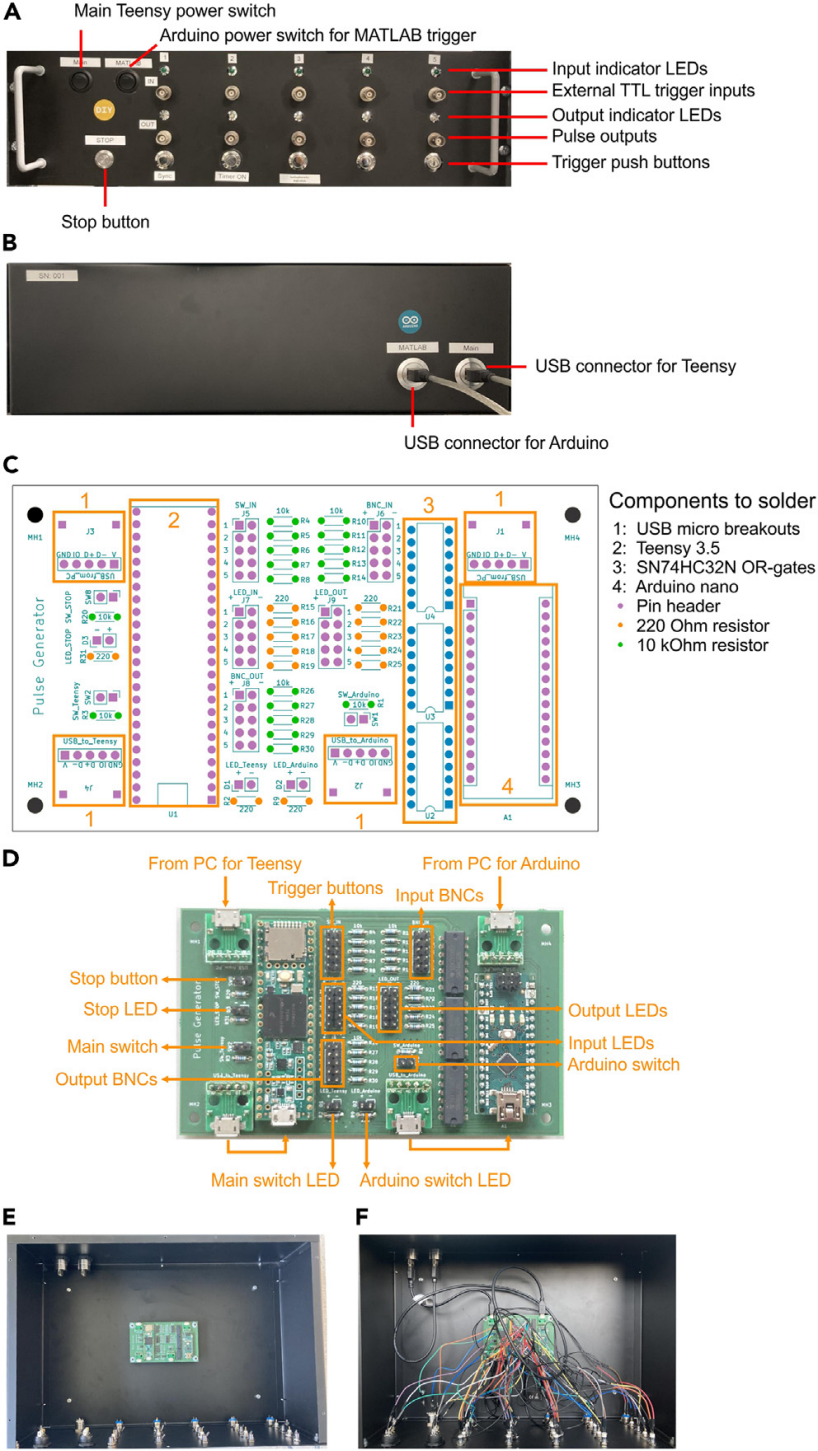
Building a custom-made pulse generator (A) An assembled pulse generator in a 19-inch rack housing. (B) The back side of the pulse generator housing. (C) Printed circuit board (PCB) of the pulse generator. Components to solder are indicated. (D) PCB with all components soldered. Connections with a computer and other components in the housing are indicated. (E) Interior of the pulse generator before connections are made. (F) Interior of the pulse generator with all connections.

There are commercially available high quality pulse generators such as Master 8 (AMPI) and Multistim 3800 (A-M systems), whereas low-cost open source pulse generators such as Pulse Pal^1^ have become available recently. In our particular case, we had specific requirements in addition to a budget limitation: (a) we needed only monophasic digital input/output; (b) we needed 5 independent channels; (c) output in each channel should be triggered by either pressing a button, external digital input, or MATLAB function. Most commercially available pulse generators do not have trigger input for each channel. Thus, we developed a custom pulse generator using Arduino and Teensy technology (Figure 1A). The Teensy board serves the main pulse generator function, whereas the Arduino board works as an interface between the Teensy board and MATLAB. This custom-designed solution offers 5 independent channels. Pulses are triggered by either external TTL input, push button, or MATLAB function via Arduino. Each of the five channels offers four different pulse modes: pulse, toggle, asinput and togglePulse (Table 1). The input is 5 V tolerant TTL, and the output is 3.3 V TTL. To streamline the building process, we designed a printed circuit board (PCB) of the pulse generator (Figures 1B and 1C).

***Alternatives:*** Commercially available pulse generators and other open-source pulse generators can be used for the synchronization technique introduced in this protocol.

1. Solder components on the PCB (Figure 1C).
2. Make the housing (Figures 1A and 1B).
  a. Install the following components in the front panel:
    i. Two power switches with LED indicators.
    ii. A push button with an LED indicator (Stop button).
    iii. 5 LEDs as input indicators.
    iv. 5 BNC female connectors as external inputs.
    v. 5 LEDs as output indicators.
    vi. 5 BNC female connectors as outputs.
    vii. 5 push buttons as trigger buttons.
  b. Install 2 USB-B/USB-A connectors in the back panel (Figure 1B).
  c. Install the soldered PCB in the bottom panel using 4x M3 screws and standoff spacers.
3. Connect the PCB to components in the housing using jumper wires and USB cables (Figures 1D–1F).

***Note:*** When connecting the LEDs, pay attention to the polarity: connect the pins with the “+” signs on the PCB to the longer wires of the LEDs. There is no polarity for switch and button connections. Since necessary resistors are already installed on the PCB, it is unnecessary to connect further resistors for LEDs and switches.

***Note:*** Panel mount power switch with LED indicator may have 3 or 4 connectors. See the datasheet of the purchased switch to confirm the role of each connector.

***Note:*** You may need to solder pin headers to connectors of switches, BNC connectors and push buttons for connections with jumper wires.

***Alternatives:*** Rack-mount housing is not necessary for the function of the pulse generator, but it substantially facilitates practicality of the operation.

4. Connect the two USB cables of the pulse generator housing to a computer (Figure 1B).

**Table 1:** Four modes of the pulse generator

Mode	Set parameter function	Description
pulse	setPulseParam(pulseWidth, interval, nPulse)	Trigger starts a pulse train
toggle	N/A	Trigger toggles between high and low output state
asinput	N/A	Output pulse is identical to input trigger
togglePulse	setTogglePulseParam(pulseWidth, interval)	Trigger toggles between running and stopping pulse train

### Pulse generator software settings (standalone)

**Timing: 20 min**

You first need to configure a custom sketch on the Teensy board to set pulse settings. This can be achieved by using the Arduino Integrated Development Environment (IDE) and uploading the provided example sketch, “myPulses.ino”, available on the github repository at https://github.com/neurogelotology/synchronization_protocol/tree/main/Pulse_Generator_software.

5. Open Arduino IDE, Sketch > Include Library > Add .Zip Library, and select “pulseGen.zip”.
6. Turn on the main switch of the pulse generator.
7. Setup connection of the Teensy board.
  a. Click on “Tools” in the main menu and select “Port” from the drop-down list.
  b. A list of available serial ports will appear. The Teensy should be listed as “Teensy” followed by the PORT number (e.g., “Teensy (COM3)”).
  c. Select the correct PORT.

***Note:*** If the Teensy is not listed, try turning off the pulse generator and turning it on.

***Note:*** If multiple PORT numbers are listed and you are not sure which is the Teensy PORT, turn off the pulse generator main switch. Click on “Tools” and select “Port”. Note which PORTs are listed. Turn on the pulse generator main switch and view the list of the PORTs again. The newly added PORT is the one that Teensy is connected to.

8. Click on “Tools” in the main menu, and from Board list, select “Teensy 3.5”.
9. File > Open, select “myPulses.ino” file.
10. Define a pulse mode for each channel in setup() function. Pulse modes must be either “pulse”, “toggle”, “asinput”, or “togglePulse” (Table 1). For example:

ch2.mode = “pulse”;

11. For channels with “pulse” mode, set pulse parameters using the method:

setPulseParam(pulseWidth, interval, nPulse)

where pulseWidth and interval are in [ms].

ch2.setPulseParam(50, 100, 10);

12. For channels with “togglePulse” mode, set pulse parameters using,

setTogglePulseParam(pulseWidth, interval)

method in [ms].

13. Click on the check icon on the top left “Verify”, then click on the right arrow icon next to it “Upload”.

***Note:*** We use 1 Hz pulse trains in Ch1 as the synchronization TTL in this protocol. Therefore, the following settings are needed:

ch1.mode = “togglePulse”;

ch1.setTogglePulseParam(50, 1000);

**CRITICAL:** Modify only parameters in the,

void setup(){}

***Note:*** To ensure a successful verification and upload process, make sure that the Teensyduino is installed on your computer. Additionally, check that the correct PORT and Board for the Teensy board are selected in the Arduino IDE settings (see step 7). If you are still encountering issues, try restarting the software and the pulse generator.

***Optional:*** If you need to frequently switch between different pulse generator settings, you can create multiple versions of the Arduino sketch file (.ino) by making copies of “myPulses.ino” with different names. You can then easily change the pulse generator settings by uploading the desired .ino sketch file that corresponds to the specific settings you require.

14. Test the pulse generator by pressing trigger buttons.

***Note:*** The output pulses are indicated by the output LED on each channel. Pressing the STOP button will stop all running pulses.

***Optional:*** You can monitor the pulse output using an oscilloscope. To do so, connect the oscilloscope probe to the BNC output connector on the pulse generator. You can then observe the pulse frequency and the voltage level on the oscilloscope. This will help you verify that the pulse generator is functioning correctly and outputting the desired pulses.

### Pulse generator software settings (MATLAB trigger)

**Timing: 3 min**

With MATLAB integration, users can design more flexible trigger patterns for the pulse generator. Specifically, MATLAB triggers a pulse output in the Arduino in the pulse generator, which in turn trigger a channel of the main Teensy board. The pulse generator can be controlled using four MATLAB functions (Table 2; available at https://github.com/neurogelotology/synchronization_protocol/tree/main/Pulse_Generator_software).

***Note:*** In our pulse generator, [D2, D3, D4, D5, D6] output pins on Arduino are wired to inputs of channels 1-5 of the pulse generator respectively.

15. Turn on both the main and Arduino power switches on the pulse generator (Figure 1A).
16. Restart MATLAB.
17. Type.

myArduino = connectArduino();

**Table 2:** MATLAB functions to control the Arduino to trigger the pulse generator channels

Function	Description
a = connectArduino()	Generates an Arduino instance. e.g., myArduino = connectArduino();
offPin(a, outpin)	Makes TTL state of the specified pin LOW. e.g., offPin(myArduino, ‘D2’);
onPin(a, outpin)	Makes TTL state of the specified pin HIGH. e.g., onPin(myArduino, ‘D2’);
trgPulse(a, outpin)	Generates 1 pulse with 100 ms duration in the specified pin. e.g., trgPulse(myArduino, ‘D2’);
exampleTimer()	A simple example use of MATLAB triggers of the pulse generator. “Start” button starts the timer and sync pulse, and stimulation (when the pulse generator Ch2 is connected to e.g., stimulus isolator or LED driver) is triggered at certain timings.

in the command window and press Enter. It should display “Nano 3 is connected” in the command window.

**CRITICAL:** If connectArduino does not generate an Arduino instance, please refer to problem 1.

18. Try executing,

trgPulse(myArduino, ‘D2’);

which sends a pulse to the Ch1 input of the pulse generator. If you configured the togglePulse mode in the previous preparation, the Ch1 output LED should start blinking at 1 Hz.

**CRITICAL:** Since output of the Teensy 3.5 board is 3.3 V, the pulse generator may not be able to trigger devices that requires 5 V TTL input. It is possible to convert 3.3 V TTL to 5 V TTL using a level shifter circuit, which, however, might cause delays in the timing of the pulses.

***Optional:*** In addition to signal synchronization, the pulse generator can be used for a variety of applications including triggering simulations in a stimulus isolator, triggering optogenetic stimulations, switching environmental illumination, and activating audio visual stimuli. Each channel of the pulse generator works independently. For instance, users can design specific behavioral paradigms where optogenetic stimulations are scheduled at certain timings. In such cases, the pulse generator can be used to trigger optogenetic stimulations, and the timing of the stimulations can be controlled in MATLAB. Example MATLAB function to trigger sync pulses and stimulations is available at https://github.com/neurogelotology/synchronization_protocol/blob/main/Pulse_Generator_software/exampleTimer.m.

### Hardware settings

**Timing: 1–3 h**

**Figure 2:**
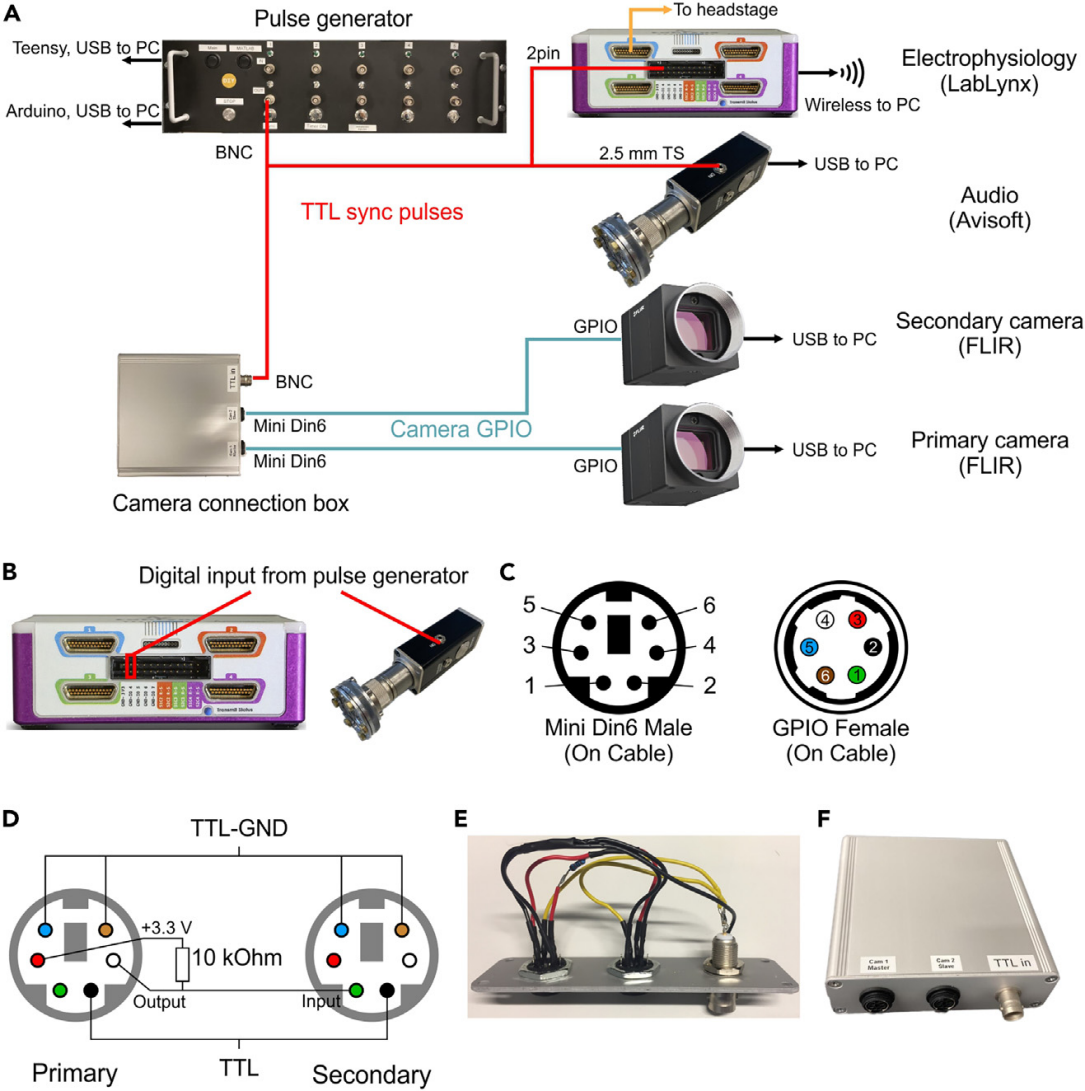
Hardware settings (A) Overview of the hardware connections. The pulse generator sends sync TTL pulses to the electrophysiology device, audio device, and the camera connection box. Two cameras and the camera connection box are connected using camera GPIO cables. (B) Digital input connectors of the LabLynx (Neuralynx) electrophysiology device and the Avisoft microphone. (C) Pinouts of the Mini Din6 male connector (left) and the GPIO female connector (right). The wires of the cables should be soldered to the corresponding wires. (D) Diagram of wiring between the Mini Din6 female sockets for the primary and the secondary cameras in the camera connection box. The diagram is a view from the breakout pin side (inside the connection box). (E) Wired connectors. (F) Assembled camera connector box.

The goal of this section is to establish connections between all devices. To demonstrate this process, we use LabLynx Portable System (Neuralynx) for electrophysiological recordings, UltraSoundGate 116Un (Avisoft) for audio recordings, and two BFS-U3-28S5C-C cameras (FLIR) for behavioral videography. A comprehensive overview of the device wiring is depicted in Figure 2A.

19. Connect Ch1 output of the pulse generator to the digital I/O connector on LabLynx (Figure 2B, left) using a BNC-AL 0.64 socket cable. The black GND socket should be connected to the lower pin of the LabLynx.
20. Construct a cable with a BNC male connector on one end, and a 2.5 mm TS connector on the other.
  a. Cut a BNC male cable and a 2.5 mm TS cable in the middle.
  b. Strip the insulation of the two cables. Separate the core wire and the shield wires.
  c. Insert heat shrink tubes on one of the cables and the core wire.
  d. Solder the core wires of the two cables together, and apply a heat gun to the heat shrink to ensure proper insulation of the core wire.
  e. Solder the shield wires of the two cables together.
  f. Apply a heat gun to the heat shrink covering the cable.
21. Test connectivity using a multimeter.
  a. Set the multimeter to continuity mode, which is typically with a diode symbol with an audio feedback feature. In continuity mode, the multimeter will beep if there is an electrical connection between the two test probes.
  b. Test the connection between the BNC pin and the tip of the TS jack. The multimeter should beep if the connection is good.
  c. Next, test the connection between the BNC shield and the sleeve of the TS jack. The multimeter should beep if the connection is good.
  d. Finally, test the insulation between the BNC tip and the sleeve of the TS jack. There should be no beep, indicating that there is no electrical connection between these two points.

**CRITICAL:** Whenever constructing a custom cable using soldering, it is always recommended to test the connection.

22. Connect Ch1 output of the pulse generator to the DIN socket on the UltraSoundGate using the BNC-TS cable (Figure 2B, right). BNC distributors (T- or Y-shaped) can be used to split the pulse signals from the pulse generator.
23. Construct two cables that have a GPIO (Hirose HR10) connector on one end and a mini din6 male connector on the other.

***Note:*** Ensure the wires are soldered properly to the corresponding pins of each connector, as depicted in Figure 2C. See steps 20-21 for soldering, insulation and testing the connections.

24. Assemble a camera connection box housing with two panel mount mini din6 female sockets and a BNC female connector, and solder the wires to the appropriate pins according to the wiring diagram (Figures 2D–2F).
25. Connect the camera connection box and two cameras using the GPIO-Mini Din6 cables, and connect the camera connection box and the pulse generator Ch1 output with a BNC cable (Figure 2A).

***Note:*** The camera connection box that serves as a hub for managing signals from the two cameras and the pulse generator. In specific, using the connection box, pulses from the pulse generator are sent to both cameras. Moreover, exposures between the two cameras can be synchronized. The diagram in Figure 2D provides a visual representation of the wiring in the connection box. Here we use commercially available mini din6 cables to interface between the camera connection box and the GPIO (General Purpose Input/Output) cables that connect to the cameras (Figure 2C).

***Alternatives:*** Soldering all connections directly may work, but having a connection box makes the setup more organized and convenient to use in practical settings. The connection box allows for a clear and easy way to connect and disconnect the cameras and the pulse generator. Additionally, having a connection box can make the setup more portable, which can be easily disconnected and transported to a different location.

**CRITICAL:** Having synchronized capture is crucial for accurate multi-camera video analysis, as it ensures that the exposure timing of both cameras is perfectly aligned.

***Note:*** The cameras used in this setup offer triggered exposure, meaning that an exposure of the primary camera will trigger an exposure of the secondary camera, thereby ensuring that the exposures are synchronized. For technical details about synchronized capture, readers are encouraged to consult the blog post by FLIR.

**CRITICAL:** GPIO connections (Figure 2D) for synchronized exposure and logging external TTL input may vary between camera models and camera manufacturers. To ensure proper wiring of the connection box, it is crucial to refer to the specific details provided in the user manual of the camera being used.

***Alternatives:*** Signal synchronization of video using a camera that does not accept external TTL input such as a webcam or a camcorder can be achieved by placing an LED visible to the camera. The anode (long pin) of the LED should be connected to the core pin of the pulse generator’s Ch1 output, while the cathode (short pin) should be connected to a 220 Ohm resistor which in turn should be connected to the shield of the pulse generator’s Ch1 output. The LED method for camera synchronization has limitations in terms of sensitivity to ambient lighting conditions, and complexity in ensuring a clear field of view, making it less suitable for applications involving challenging lighting conditions or multiple cameras.

### Camera software settings (SpinView)

**Timing: 20 min**

In this section, we demonstrate how to configure the settings of the camera software. Specifically, we use the SpinView software for two BFS-U3-28S5C-C cameras by FLIR as an example.

**CRITICAL:** Settings may vary between camera models and camera manufacturers. For the details of camera settings, consult the user manual of the camera.

26. Connect cameras to the USB3.1 interface.
27. Run SpinView software.
28. Confirm the two cameras are listed under “Devices” and can capture images by pressing the “Play” icon.

**CRITICAL:** The latest Spinnaker SDK may not be compatible with the bonsai software, which we use in the next section. We confirmed that Bonsai.Spinnaker version 0.7.0 is compatible with Spinnaker SDK version 1.29.0.5. Later version of Spinnaker SDK leads to crash of bonsai upon recording.

***Note:*** For further details of the SpinView software, see the official manual by FLIR.

29. Configure settings for synchronized exposure on the primary camera.
  a. To select a camera as the primary, go to the “Devices” section and click on the desired camera.
  b. In the properties section below, click on the “Features” tab at the bottom.
  c. Expand “Digital IO Control”.
  d. For “Line Selection”, select “Line 2”.
  e. Enable “3.3 V Enable”.
30. Configure settings for the secondary camera.
  a. Click on the secondary camera in the “Devices” section.
  b. In the properties section below, click on the “GPIO” tab at the bottom.
  c. For “Trigger Source”, select “Line 3”.
  d. For “Trigger Overlap”, select “Read Out”.
  e. For “Trigger Mode”, select “On”.
31. Save these settings as a user setting.
  a. Select the primary camera in “Devices”.
  b. From the “Features” tab, Blackfly S, open “User Set Control”.
  c. For “User Set Selector”, select “User Set 0”.
  d. For “User Set Default”, select “User Set 0”.
  e. On “User Set Save”, click on “Execute”.
  f. Select the secondary camera in “Devices”.
  g. Repeat b-e but with “User Set 1”.
32. Verify that the secondary camera’s exposure is triggered only when the primary camera is actively capturing images.

### Video recording software settings (bonsai)

**Timing: 30 min**

This section describes the use of the bonsai software^2^ for video recordings. Bonsai provides a visual language for reactive programming and access to FLIR cameras through the Spinnaker library. The example “TwoCam.bonsai” is available at https://github.com/neurogelotology/synchronization_protocol/tree/main/Bonsai. We introduce three custom features on this setting. First, frame ID is embedded on each frame of the videos as an image. This helps users notice if there are drop frames, or frame mismatches between the two cameras during analysis. Second, timestamp of each frame exposure as well as sync TTL state upon exposure are collected from the cameras and saved as CSV files. Since actual frame rate in the camera is not perfectly constant in every frame, exposure timestamps information increases accuracy of video analysis. Third, number of drop frames and sync TTL pulse can be monitored real time so that users can notice when the system is not working properly.

**Figure 3:**
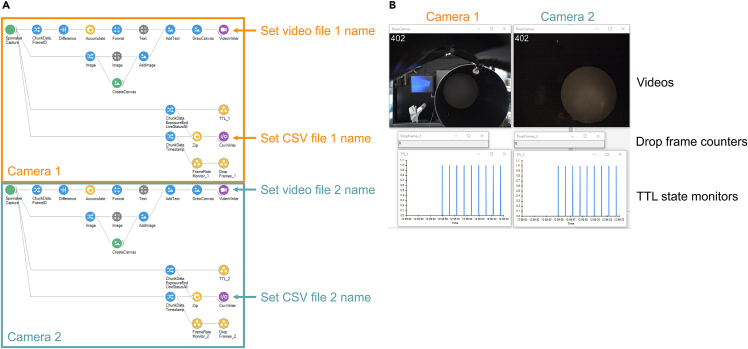
bonsai configuration to manage synchronization The TwoCam.bonsai file manages to record videos from two FLIR cameras, and save frame timestamps and TTL states for each camera. (A) Screenshot of the TwoCam.bonsai program. The users need to set file names of the videos and the CSV files before recording. (B) Screenshot of a video recording with TwoCam.bonsai. For each camera, a video monitor (frame numbers are embedded at the top left corner), a drop frame counter, and a sync TTL pulse state monitor are shown.
35. Set file names (Figure 3A).
  a. Click on the top right “VideoWriter” node with a camera image and purple background.
  b. In the Properties panel at right, click “FileName” and click on the “...” button on the right.
  c. Navigate the destination folder and type the primary video file name that ends with “.avi”.
  d. Next, click on the purple “I/O” node below the primary camera node.
  e. Similarly, set the file name for the timestamp file that ends with “.csv”.
  f. Similarly, set the video file name and the timestamp file name for the secondary camera below.
36. To ensure that both cameras are capturing images correctly, click “Start” in the top left (Figure 3B).

***Note:*** Verify that the frame ID is indicated on each image. Additionally, if the pulse generator is in operation, the TTL state should be displayed in real time. If the interval between frames is longer than double of the expected inter-frame interval, it will be counted as a dropped frame.

33. Open Bonsai.
34. File > Open, then select TwoCam.bonsai file (Figure 3).

**CRITICAL:** If bonsai crashes upon “Start”, the Spinnaker SDK version may not be compatible with the Spinnaker Library of bonsai. Try installing different Spinnaker SDK versions.

**CRITICAL:** If the dropped frame counter keeps increasing, refer to problem 2.

### Audio recording software settings (Avisoft RECORDER)

**Timing: 10 min**

Here we focus on the settings regarding the TTL sync pulses in Avisoft RECORDER software. In contrast to video recordings where the timestamp and TTL state of each frame are saved in a separate CSV file, Avisoft RECORDER saves external TTL states in the least significant bit (LSB) of the audio signal within the .wav file. Official manual of the RECORDER can be found here.

***Note:*** Avisoft RECORDER offers various options for handling external TTL inputs, such as triggering recording start with a TTL pulse. In this demonstration, we will manually start and stop the recording without using the triggered record mode.

37. Connect an Avisoft device to a computer.
38. Open Avisoft-RECORDER software. If the software is not launched because the driver is not found, see problem 3.
39. Right click on the RECORDER window to open Configuration.
40. From the “Trigger” drop down, select “UltraSoundGate DIN”.
41. Disable “Toggle”, disable “TC”, and enable “!”.
42. From the “Trigger” drop down, select “permanent (unlimited)”.
43. From the “Input Device Settings” section, click on the “Settings” button.
44. Enable “Show DIN”.

***Note:*** With this configuration, the external TTL pulses from the pulse generator will not trigger anything, but will be saved in the LSB of the .wav file. Furthermore, the external TTL states will be indicated as blue bars on the top of the spectrogram.

### Recording experiment

**Timing: Depends on experimental paradigm (a few minutes to hours)**

**Figure 4:**
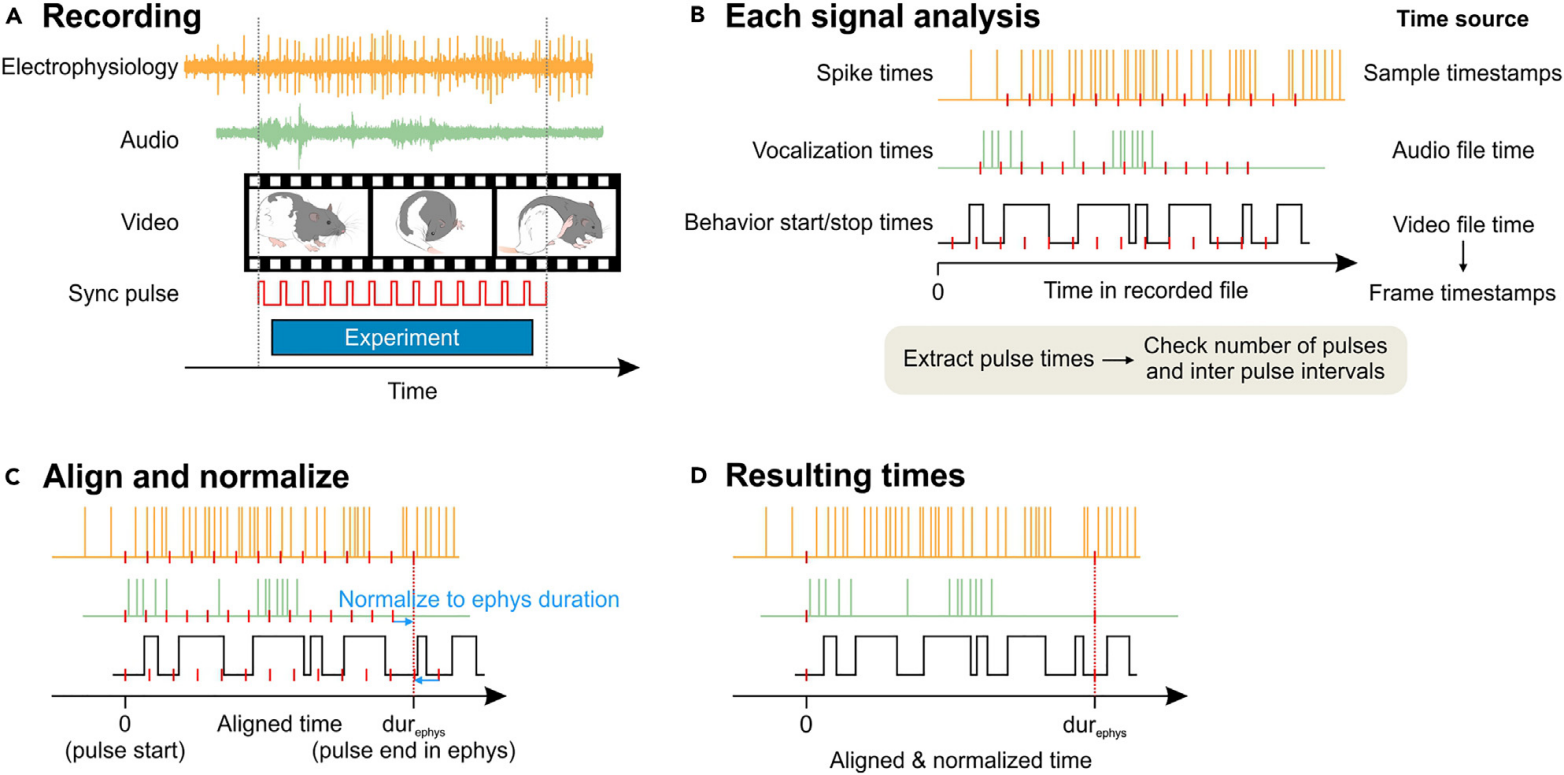
Workflow of the signal synchronization (A) A recording session yields electrophysiology data (yellow), audio data (light green), and video data (film). While acquiring signals from these devices, sync pulse train (red) is sent to the devices from the pulse generator. Experiment is performed while the sync pulse train is being sent. (B) After the recording session, each signal will be analyzed to acquire time vectors of events. Spike sorting is performed to acquire spike times from the electrophysiology data. Vocalization analysis is performed to acquire vocalization times from the audio data. Start and stop times of behaviors are detected in the video analysis. Moreover, sync pulse times (red ticks) are extracted in each signal. To ensure that all the sync pulses were saved in each signal, compare the number of pulses and inter-pulse intervals between the signals (see Figure 4). (C) The time of the first pulse is subtracted from the time vectors of events in each signal to align the start time. Then, normalize the duration of each signal (time between the first and the last pulse) to the duration of one of the signals with high temporal precision (e.g., electrophysiology). (D) Resulting time vectors are aligned and normalized.

We will perform recording of electrophysiology, ultrasound and videos, while sending a synchronization TTL to the recording devices (Figure 4A).

45. Open Cheetah (Neuralynx) software. Click on the “ACQ” and the “REC” to start recording electrophysiology.
46. Open Avisoft RECORDER (Avisoft) software. Click the window to start recording audio.

***Note:*** If the software is not launched because the driver is not found, see problem 3.

47. Open bonsai software. Set four file names as described in the step 35 of the preparation, and click the “Start” button to start recording videos.
48. Press the Ch1 trigger button of the pulse generator to start the sync pulse train. Verify that each device is properly receiving the TTL pulses.
  a. On Cheetah, new TTL events are listed in the “Events” window.
  b. On Avisoft RECORDER, the TTL status is indicated by blue lines at the top of the spectrogram.
  c. On bonsai, the TTL status is plotted on a separate window for each camera (Figure 3B).
49. Start experiment e.g., behavioral paradigm.

**CRITICAL:** Throughout the recording session, it is important to ensure that the TTL pulses are being delivered to each device and that the drop frame counters in bonsai are not increasing. Monitoring these parameters can help identify issues and prevent data loss. If you encounter a continuous increase in the drop frame counters, refer to problem 2 for a possible solution.

50. After completion of the experiment, press the Ch1 trigger button of the pulse generator to stop the sync pulse train.
51. Stop recording on each device.

**CRITICAL:** Sync pulse train must start only after all the devices start recording, and stop before any device stops recording to ensure that the same number of pulses are recorded in all devices (Figure 4A).

### Signal alignment

**Timing: 30 min after signal analysis**

After a recording session has finished, each signal is analyzed typically to produce time vectors or continuous time series (Figure 4B). For example, extracellular electrophysiological signals can be analyzed using spike sorting software to produce spike times for multiple single units. Audio analysis can yield time points of vocalizations. Behavioral analysis of video data can produce start and stop times for different behavioral events. Furthermore, times of the sync pulses in each signal will be extracted as a time vector. In this section, we demonstrate how to align time vectors resulted from different devices.

52. Complete analysis of each signal.

***Note:*** We do not cover each signal analyses in this protocol. The results are expected to be time vectors or continuous time series that may accompany additional data. For example, the electrophysiology signal results in “spike_time” and “unit_id” arrays for multiple neuronal units. Audio analysis may yield “vocalization_time” and “vocalization_type” arrays. Video analysis can result in “event_start”, “event_end” and “event_name” arrays. To be consistent with later analyses, we expect that times are in [s]. Typically, the time values in the resulting time vectors are either timestamps of the recording device, or time relative to the start of the acquisition, dependent on analysis software. In our case, we use JRClust^3^ for spike analysis, DeepSqueak^4^ for vocalization analysis, and BORIS^5^ for video analysis.

53. Extract time points of the sync pulses from each signal. Execute,

nlxTTLtime = getNlxTTL_demo();

and select the Events.nev file of the recording session.

***Note:*** We provide example data and a MATLAB Live Script for the sync pulse analysis available at https://github.com/neurogelotology/synchronization_protocol/blob/main/syncTTL/sync_pulse_analysis.mlx.

***Note:*** The Neuralynx device stores timestamps of digital inputs in the “Events.nev” file. We provide a MATLAB function “getNlxTTL_demo.m”, which extracts a sync pulse rise time vector, as well as acquisition start time as timestamps of the Neuralynx device in [s].

***Note:*** Event ID for the digital input in the Events.nev file may vary between Neuralynx devices. To check the event ID for the digital input of your Neuralynx device, download and install NeuraView. Open the Events.nev file on NeuraView. Press Ctrl+D to show the data details window. From the drop down menu, select the Events.nev file. Click on the “Record Data” tab. It will show a table of all events including “Starting Recording”, and “TTL Input”. Check the “ID” of “TTL Input” events. In the getNlxTTL_demo.m file, change the value in the line

TTLeventID = 21;

to the ID of your device.

54. Execute,

audioTTLtime = getAudioTTL_demo();

and select the .wav file of the recording session.

***Note:*** The Avisoft device stores the external TTL status in the least significant bit (LSB) within the .wav file. We provide a MATLAB function “getAudioTTL_demo.m” to extract a sync pulse rise time vector as audio file time in [s].

55. Execute,

cam1TTLtime = getVideoTTL_demo();

and select the .csv file of camera 1 of the recording session. Repeat the same for camera 2.

***Note:*** With the configuration described above, bonsai saves timestamp and TTL state of each frame in a CSV file for each camera. We provide a MATLAB function “getVideoTTL_demo.m” to extract a sync pulse rise time vector as timestamp of the camera in [s]. Furthermore, “getVideoTime_demo.m” extracts a time vector of all frames as timestamp of the camera in [s].

56. Verify the sync pulses in each device. This involves checking whether the numbers of pulses are equal across all devices, and whether the inter-pulse intervals are stable.

**Figure 5:**
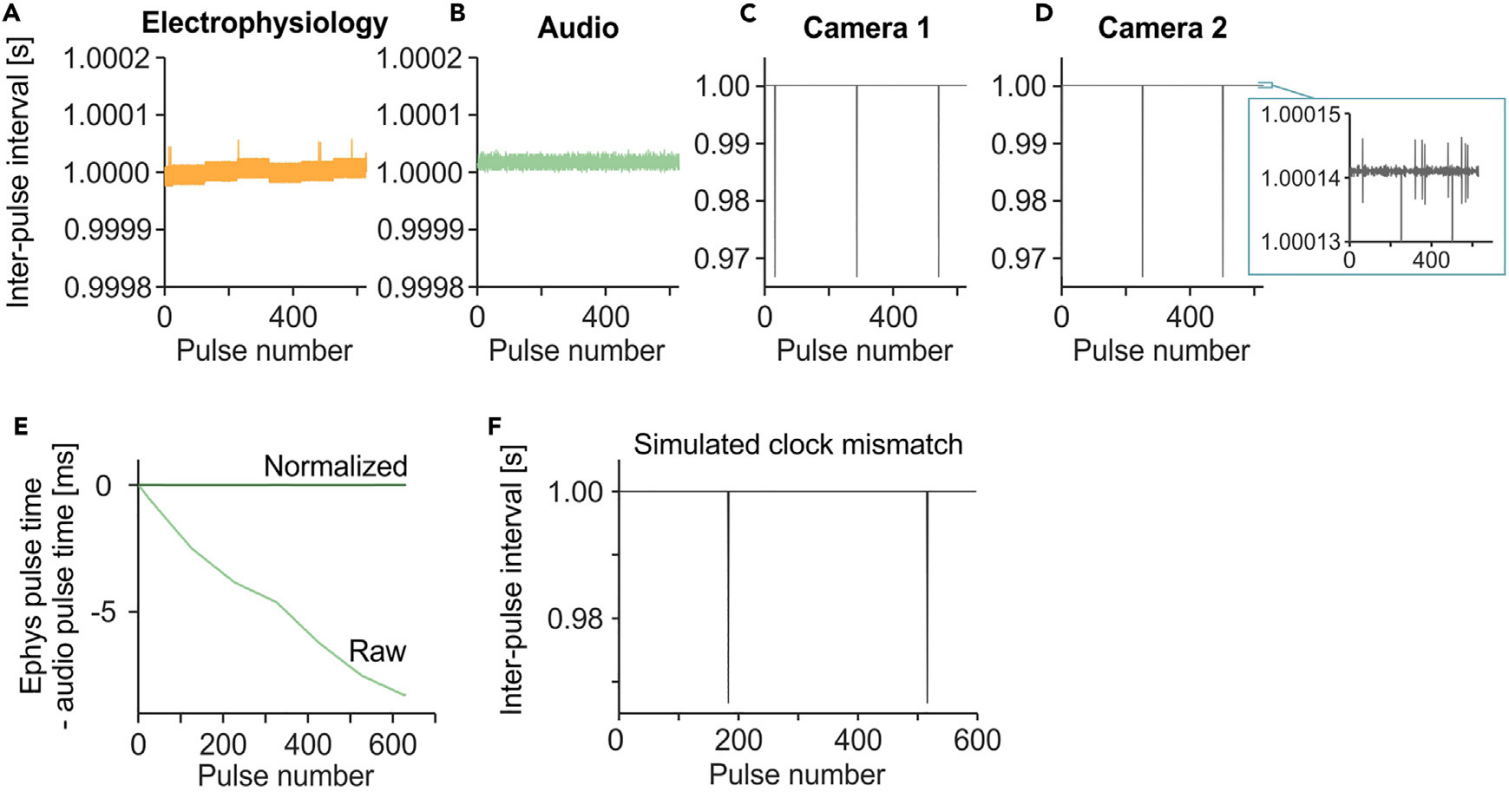
Sync pulse analysis in a representative recording session (A) Inter-pulse intervals of 1 Hz sync pulses in the electrophysiology device timestamps across the recording session. (B) Same as (A) but in the audio device. (C) Same as (A) but in the camera 1. (D) Same as (A) but in the camera 2. Inset shows smaller y-axis range. (E) Difference in each sync pulse time between electrophysiology and audio device before (‘Raw’) and after (‘Normalized’) normalization of the session duration. (F) Simulated inter-pulse interval of a camera where the camera clock runs 0.01% slower than the pulse generator clock, closely matching the actual data in (C) & (D).

**Table 3:** Example of sync pulse verification

Device	Number of pulses	Durations (last pulse time - first pulse time) [s]	Mean inter-pulse interval [s]	Minimum inter-pulse interval [s]	Maximum inter-pulse interval [s]
electrophysiology	630	629.0010	1.0000	1.0000	1.0001
audio	630	629.0093	1.0000	1.0000	1.0000
camera 1	630	628.9887	1.0000	0.9668	1.0001
camera 2	630	628.9887	1.0000	0.9668	1.0001

***Note:***Figures 5A–5D and Table 3 show verification of sync pulses in an example recording session. Further details of the sync pulse analysis are described in overview of the sync pulse analysis.

57. Align the time vector data from different signals such as spike times, vocalizations times and event times, using the sync pulses. Alignment of the signals can be achieved by subtracting the time of the first sync pulse from all time vector data for each device, resulting in time vectors relative to the first sync pulse time (Figure 4C).

***Note:*** Time values in the time vector data can be either as timestamps of the recording device, or times relative to the first sample time of the recording, dependent on the analysis algorithm. In the latter case, difference between the first sample timestamp and the first sync pulse rise timestamp must be subtracted from the time vector data.

58. Normalize the session duration of each time vector to the session duration of the electrophysiology data. Normalization of the duration can be achieved by dividing the time vector data from a given device by the session duration (last sync pulse time - first sync pulse time) of that device, and then multiplying by the session duration of a reference device (Figures 4C and 5E). $\frac{times}{dur}\times ref_{dur}$. In our demonstration, we use the electrophysiology device as a reference. For technical details of this procedure, refer to normalization of the session durations.

## Expected outcomes

Using the signal synchronization methods we have introduced in this protocol, signals recorded in different devices are temporally aligned (Figure 4D). Temporally aligned data can be used to plot peristimulus time histogram to analyze relationships between different signals, such as neuronal firing rate or vocalization rate upon a behavioral onset.^6^^,^^7^^,^^8^^,^^9^

## Quantification and statistical analysis

### Overview of the sync pulse analysis

We evaluated the performance of our custom pulse generator by analyzing the sync pulses recorded by LabLynx, Avisoft and two FLIR cameras during a 10.5-min recording session (Table 3). All 630 sync pulses were detected in all devices, and the session duration (last pulse time – first pulse time) was on average 628.9969 ± 0.0044 s (mean ± SEM). To account for differences in session durations between devices, we describe a normalization method in the next section. Inter-pulse interval of the sync pulse, which is theoretically 1 s constantly, was stable both in LabLynx (Figure 5A) and Avisoft (Figure 5B), where the fluctuation was smaller than ±0.1 ms. The video devices periodically showed small inter-pulse intervals (Figures 5C and 5D) due to clock mismatch between the pulse generator and the cameras, which we explain in ”artifacts due to the clock mismatch” section. Overall, our protocol using the custom pulse generator offers highly precise signal synchronization between multiple recording devices.

### Normalization of the session durations

Due to differences in hardware clocks, time data from different recording devices are measured in different timescales. In other words, 1 s in one device may not have the same duration as 1 s in another device (Figures 5A and 5B). This clock difference is typically consistent over time, resulting in a linear drifting effect when the time difference between sync pulse times from different devices is plotted over pulse index (Figure 5E, “Raw”). To analyze the relationship of data recorded in different devices, this linear drifting needs to be corrected. To achieve this, session duration (last pulse time - first pulse time) recorded in each device needs to be normalized to the session duration recorded in a reference device (Figure 5E, “Normalized”). In our example, we select the electrophysiology as the reference device because of the high sampling rate (30 kHz), whereas the videos’ frame rate is 30 fps. Additionally, the time information of the electrophysiology data is given as the device’s clock timestamp. Although the audio data has a higher sampling rate of 256 kHz, the audio data time is given as the time in .wav file with a fixed (ideal) sampling rate and not the device’s clock timestamp, which could be fluctuating in reality (Figures 5A and 5B). The normalization can be performed as mentioned in the “signal alignment” section.

### Artifacts due to the clock mismatch

In Figures 5C and 5D, we present the inter-pulse intervals of sync pulses recorded in the two cameras. In addition to negligible fluctuations in the sampling rate (Figure 5D inset), the inter-pulse intervals show a periodical decrease of 0.0332 s, which is precisely the inter-frame interval of the video recordings. Through a simulation, we verified that the periodical decrease in the inter-pulse interval was caused by clock mismatch between the camera and the Teensy board of the pulse generator. In our simulation, we set the camera clock to run 0.01% slower than the clock of the pulse generator, indicating that 1 s in the camera is equivalent to 1.0001 s in the pulse generator. Additionally, we set the camera exposure time to 0.015 s for each frame, with a frame rate of 30 fps. The simulated inter-pulse interval, shown in Figure 5F, closely matches the actual data observed in Figures 5C and 5D. Consequently, we conclude that the periodical decrease in the inter-pulse interval is not indicative of frame drop, but rather a result of clock mismatch between the cameras and the pulse generator. By normalizing the duration as described above, the video data can be synchronized with signals from other devices. The simulation of the clock mismatch is available at https://github.com/neurogelotology/synchronization_protocol/blob/main/syncTTL/sync_pulse_analysis.mlx.

## Limitations

While the protocol presented in this article offers an effective solution for precise signal synchronization, there are several limitations to its applicability.

Device specificity: This protocol focuses on specific devices and software settings as examples, and may not be directly applicable to all recording setups. The general concept can be adapted to other similar devices and software, but users must verify compatibility and adjust settings accordingly. It should be noted that some recording devices such as conventional microphones and webcams are not capable of receiving digital inputs.

Data format specificity: The format of the data may vary between recording devices and analysis software. The users may need to adjust the analysis functions we provide.

Pulse generator dependency: This protocol partly relies on the use of our custom-made pulse generator for signal synchronization, which may not be applicable for all users. Alternatively, any common pulse generators can be used.

Recording environment: The accuracy of the signal synchronization depends on the stability of the recording environment including the recording devices and the computers.

Limitation of temporal precision: Our protocol may not provide the level of temporal precision required for research that demands sub-millisecond accuracy. In such cases, more advanced synchronization techniques may be necessary.

## Troubleshooting

In the Troubleshooting section, we provide solutions for common problems that can occur repeatedly during experiments. For problems that may arise during the setup phase, we have outlined potential solutions in the before you begin section and the step-by-step method details section.

### Problem 1

connectArduino does not generate an Arduino instance in MATLAB (typically occurs in pulse generator software settings (MATLAB trigger) and recording experiment).

### Potential solution

Two possible solutions:

• Turn off and turn on both main and MATLAB power switches on the pulse generator. Then restart MATLAB.
• Check COM port number of the Arduino in the device manager (Windows).
  ○ Turn off the MATLAB power switch on the pulse generator.
  ○ Open the device manager and expand the “Ports (COM & LPT)” section.
  ○ Turn on the MATLAB power switch on the pulse generator.
  ○ You should see a new COM port added to the list. Note the COM pote number.
• If the Arduino is COM3, for example, execute,

arduino(‘COM3’, ‘Nano3’)

in the Command Window of MATLAB, which uploads server code in the Arduino. This issue can happen when you first operate the pulse generator from the MATLAB, or change the USB port on the computer.

### Problem 2

The dropped frame counter on bonsai keeps increasing (typically occurs in video recording software settings (bonsai) and recording experiment).

### Potential solution

Here are some possible causes to consider:

• Overfilled memory cache: If the computer’s memory cache is full, the recording may not function properly. Stop the recording, and restart the computer.
• Storage space: Make sure to empty the storage and free up some space at least 15% of the storage capacity as a common practice.
• Too high frame rate: If the frame rate is too high for the computer’s processing power, it can lead to recording problems. Decreasing the frame rate can resolve this issue.
• Slow writing speed: If the writing speed of the storage device is not fast enough, it can lead to frame dropping. Consider using a high-speed SSD, specifically one that is not the system drive for data storage. Avoid saving on a network drive.

### Problem 3

Avisoft RECORDER fails to start because the driver is not found (typically occurs in audio recording software settings (Avisoft RECORDER) and recording experiment).

### Potential solution

Reinstall the driver (Windows).

• Uninstall the driver.
  ○ Open Device Manager.
  ○ The Avisoft device may be recognized under either “Sound, video and game controllers”, “Universal Serial Bus controllers”, or “Avisoft-UltraSoundGate” category. If it is under “Universal Serial Bus controllers”, the device name is “USB Composite Device”.
  ○ Right click on the Avisoft device, and click “Uninstall”.
• Reinstall the driver.
  ○ Once the driver is uninstalled, disconnect and connect the Avisoft device again. Windows will automatically install a driver and assign the device as “Audio Endpoint”. This driver needs to be uninstalled by right clicking and clicking “Uninstall”. Then the device will become a “USB Composite Device”.
  ○ Update driver of this device by manually locating the driver directory to the C:∖Program Files (x86)∖Avisoft Bioacoustics∖RECORDER USGH∖Drivers.

## Resource availability

### Lead contact

Further information and requests for resources and reagents should be directed to and will be fulfilled by the lead contact, Shimpei Ishiyama (shimpei.ishiyama@uni-mainz.de).

### Materials availability

This study did not generate new unique reagents.

## Data Availability

Codes and necessary files are available at https://github.com/neurogelotology/synchronization_protocol (Zenodo DOI: https://zenodo.org/badge/latestdoi/629179984). Example data files have been deposited to Mendeley Data: https://doi.org/10.17632/98rzkwy8s6.1. See the key resources table.
